# Contribution of computed tomography in the postmortem diagnosis of drowning: a systematic review

**DOI:** 10.1590/0100-3984.2025.0048

**Published:** 2026-04-23

**Authors:** Rachel Zeitoune, Raquel Martins Loureiro, Nívia Abadia Maciel de Melo Matias, Carmen Silvia Molleis Galego Miziara

**Affiliations:** 1 Department of Forensic Medicine, Bioethics, Occupational Medicine, Physical Medicine, and Rehabilitation, School of Medicine, Universidade do Estado de São Paulo (USP), São Paulo, SP, Brazil.; 2 Instituto de Medicina Legal da Polícia Civil do Distrito Federal (IML/ PCDF), Brasília, Brazil.

**Keywords:** Drowning, Postmortem imaging, Forensic imaging, Autopsy, Tomography, X-ray computed, Afogamento, Imageamento post mortem, Imageamento forense, Autópsia, Tomografia computa-dorizada por raios X

## Abstract

The postmortem diagnosis of drowning is a challenge for forensic medicine. The objective of this review was to list the findings described in reports of postmortem computed tomography (PMCT) examinations and autopsies of drowning victims, as well as to show how PMCT facilitates the diagnosis. The PubMed, Google Scholar, the Brazilian *Coordenação de Aperfeiçoamento de Pessoal de Nível Superior* (CAPES, Office for the Advancement of Higher Education) Journals, and the International Prospective Register of Systematic Reviews (PROSPERO) databases were searched. The 17 studies included evaluated a collective total of 726 bodies of drowning victims and 477 bodies of people who died from other causes. Different PMCT protocols were used, some encompassing whole-body scans and others including scans only from the skull to the pelvis, with different slice thicknesses, all without the use of contrast. The finding most commonly described was fluid in the paranasal sinuses, mastoid air cells, and airways, referred to as specific for drowning if frothy, containing dense sediment, or both. A cutoff of 37.77 HU for the density of fluid in the paranasal sinuses was suggested to characterize drowning in salt water. Paltauf spots were detected only at autopsy. Although PMCT has proven to be a useful tool in making this diagnosis, it is too early to predict whether it can replace conventional autopsy. Current limitations include the absence of established protocols, a shortage of forensic radiologists, and low availability of CT scanners at forensic medicine facilities.

## Abstract (pt)

O diagnóstico post-mortem de afogamento é um desafio para a medicina legal. O objetivo desta revisão é elencar os achados descritos na tomografia computadorizada post-mortem (TCPM) e na autópsia de afogados e mostrar como a TCPM auxilia nesse diagnóstico. Foram pesquisadas as bases de dados PubMed, Google Acadêmico, Periódicos da Fundação Coordenação de Aperfeiçoamento de Pessoal de Nível Superior e International Prospective Register of Systematic Reviews. Os 17 estudos incluídos avaliaram um total de 726 corpos vítimas de afogamento e 477 corpos mortos por outras causas. Foram realizados diferentes protocolos de TCPM, alguns abrangendo a varredura de todo corpo e outros apenas do crânio até a pelve, com espessuras de corte divergentes, todos sem uso de contraste. Os achados mais descritos foram: líquido nos seios paranasais, nas células das mastoides e nas vias aéreas, referidos como específicos para afogamento se espumoso e/ou associado a sedimento denso; sugerido um cut-off de 37,77 UH na densidade do líquido nos seios paranasais para caracterizar afogamento em água salgada. Apenas a autópsia evidenciou manchas de Paltauf. A TCPM mostrou-se uma ferramenta nesse diagnóstico, porém é cedo prever se poderá substituir a autópsia convencional. Dentre os limites atuais destacam-se a ausência de protocolos estabelecidos, escassez de radiologistas forenses e baixa disponibilidade de tomógrafos nos institutos médicolegais.

## INTRODUCTION

Death by drowning is common worldwide. According to estimates from the World Health Organization, there are approximately 300,000 drowning deaths every year^**(1)**^. Drowning disproportionately affects children and young people, representing the fourth leading cause of death in the 1-to-4-year age group and the third leading cause in the 5-to-14-year age group^**(1)**^. It can occur in salt water (seas and oceans) and fresh water (rivers, lakes, wells, etc.).

Determining whether drowning was the cause of death for a body found in water is imperative in forensic investigations because a body submerged in water may signify secondary drowning (homicide) rather than primary (accidental) drowning. Making that diagnosis is often difficult because of the absence of definitive criteria in the autopsy, in which there can be nonspecific macroscopic findings common to other causes of death, such as the following^**(2-^4^)**^: frothy fluid in the airways; fluid accumulation in the paranasal sinuses; pleural effusion; congested, hyperinflated lungs; hemorrhage in the middle ears; fluid in the stomach; and reduced spleen weight. The detection of diatoms in the bodies, through microscopic and DNA analysis, as well as the analysis of electrolytes in the pleural fluid, can be useful in diagnosing drowning and determining whether it occurred in salt water or fresh water. However, because of postmortem changes (transformative phenomena) and contamination, those signs are nonspecific^**(5)**^. Therefore, when performed, an autopsy does not allow reliable differentiation between death by accidental drowning and a violent death with subsequent submersion of the body in water, as well as the type of water in which the drowning occurred.

With the evolution of technology, the advent of multidetector computed tomography (CT) scanners, the development of workstations capable of reconstructing three-dimensional images from the raw data acquired, and the greater availability of imaging methods in forensic medicine institutes, forensic radiology has gained importance and has come to play a larger role, as an adjunct to autopsy, in the practice of forensic medicine^**(6)**^.

The objective of this review was to list the findings described in reports of PMCT examinations and autopsies of drowning victims, as well as to show how PMCT can facilitate the pathology-based diagnosis by conventional autopsy. Given that there have been few studies of postmortem imaging in drowning victims, this study has educational importance by providing an outline to forensic physicians and radiologists who are not familiar with forensic imaging.

## MATERIALS AND METHODS

### Eligibility criteria

This systematic literature review included studies that answered the research question according to the population–intervention–comparison–outcome strategy, namely, what is the contribution of CT scans in the postmortem diagnosis of drowning. Original articles available in Portuguese and English, published between 2000 and 2024, were selected, taking into account that prior to this period the quality and availability of CT scans were limited. Meta-analyses and systematic reviews were excluded, as were case series with only three or fewer cases and case reports. We also excluded studies involving the use of artificial intelligence, because they use a method based on deep learning, whose particularities make the comparison unequal and fall outside the scope of this review.

### Article selection

**Figure 1:**
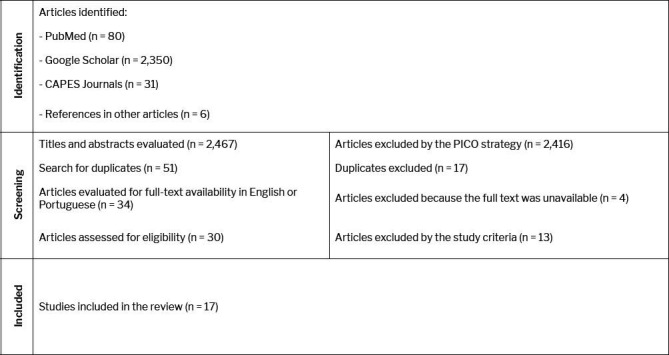
Flow chart of the article selection process, conducted in accordance with the 2020 Preferred Reporting Items for Systematic Review and Meta-Analysis statement. PICO, population–intervention–comparison–outcome.

The searches and article selection were conducted in accordance with the 2020 Preferred Reporting Items for Systematic Review and Meta-Analysis statement guidelines, as shown in Figure 1. The searches were performed between August 2024 and January 2025 in four open-access databases—PubMed, Google Scholar, CAPES Journals, and PROSPERO—using the following expressions and Boolean connectors: “virtual autopsy” AND “drowning”; “post mortem computed tomography” AND “drowning”; and “computed tomography” AND “drowning” AND “autopsy”.

The selected articles were organized in the Mendeley Library, and duplicate materials were then removed. Subsequently, a search for full-text articles was initiated; those for which the full text was not available in English or Portuguese were excluded. A search was also conducted for references cited in the articles (using the snowball technique).

Using the methods described above, we initially identified 2,467 articles. Of those, only 17 were included in the review.

### Data collection and strategy for data synthesis

Two of the authors, working independently, reviewed the selected material, evaluating titles and abstracts, with disagreements being resolved by a third author. The full texts of the included studies were analyzed, and the data were transferred to tables standardized by the authors, with the following fields: authors; year of publication; country of origin; study design; population; time of autopsy and PMCT scan; sample characteristics of drowning versus non-drowning cases; CT scan specifications and examination protocol; autopsy findings, PMCT findings, and comparison between the two; results; and study limitations.

## RESULTS

### Characteristics of the studies included in the systematic review

The 17 articles included in this review date from 2007 to 2024, with the majority having been published from 2012 onwards. All of the work was carried out in countries in the northern hemisphere, with Japan pre-dominating (with 6 articles).

Table 1 shows the characteristics of the 17 studies included. Together, they evaluated a total of 726 bodies of drowning victims and 477 bodies of victims of deaths from other causes, with a wide age range (1–100 years). Three studies^**(7–^9^)**^ did not compare groups of drowned and non-drowned individuals, focusing instead on describing the findings typical of drowning, comparing PMCT with autopsy.

**Table 1:** —Characteristics of the study populations.

Authors	Sample size		Age (years)	
	Drowning	Non-drowning	Drowning	Non-drowning
	N (sex)	N (sex)	Mean/Median (range)	Mean/Median (range)
Levy et al.^**(18)**^	28 M	12 M	24.2^*^ (19–35)	50.8^*^ (40–66)
Christe et al.^**(2)**^	10 (8 M/2 F)	20 (14 M/6 F)	52^†^ (13–81)	43^†^ (19–73)
Kawasumi et al.^**(4)**^	39 (n.d.)	112 (n.d.)	39^*^	60^*^
Kawasumi et al.^**(3)**^	38 (28 M/10 F)	73 (38 M/35 F)	67.5^†^ (19–88)	69^†^ (16–94)
Ambrosetti et al.^**(11)**^	6 (4 M/2 F)	16 (14 M/2 F)	46^*^ (18–65)	52^*^ (1–78)
Van Hoyweghen et al.^**(17)**^	14 (n.d.)	11 (n.d.)	n.d.	n.d.
Usui et al.^**(7)**^	92 (54 M/38 F)	N/A	65.4^*^ (44–90)	N/A
Plaetsen et al.^**(12)**^	41 (28 M/13 F)	9 (7 M/2 F)	58^*^ (15–90)	40^*^ (24–59)
Kawasumi et al.**(8)**	93: 22 (12 M/10 F in salt water); 71 (50 M/21 F in fresh water)	N/A	60.3^*^ (35–87) in salt water; 64.6^*^ (19–100) in fresh water	N/A
Leth and Madsen^**(15)**^	40 (25 M/15 F)	80 (50 M/30 F)	55^*^ ± 17^§^ (n.d.)	55^*^ (±17 years)
Mishima et al.^**(13)**^	77 (51 M/26 F)	50 (n.d.)	69.3^*^ (28–92)	n.d.
Gotsmy et al.^**(19)**^	55 (40 M/15 F)	35 (19 M/16 F)	45.3^*^ (1.1–87)	52.3^*^
Sugawara et al.^**(10)**^	37: 25 (13 M/12 F in fresh water); 12 (5 M/7 F in salt water)	24 (15 M/9 F)	66^*^ (55–77) in salt water; 73.1^*^ (43–95) in fresh water	60.3^*^ (22–89)
Jian et al.^**(14)**^	6 (3 M/3 F)	12 (n.d.)	n.d. (21–54)	n.d.
Kakimoto et al.^**(5)**^	52 (33 M/19 F)	59 (42 M/17 F)	67^*^ (21–91)	62^*^ (24–99)
Heo et al.^**(9)**^	54 (n.d.)	N/A	56^*^ (n.d.)	N/A
Tyr et al.^**(16)**^	44 (38 M/5 F/1 of unknown sex)	1 (M)	43^*^ (7-89)	n.d.

M, male; F, female; n.d., no data; N/A, not applicable (no control group).

* Mean.

† Median.

§ Standard deviation.

The causes of death other than drowning covered in the studies include the following: atherosclerotic coronary artery disease; aortic dissection; cardiovascular failure; sudden cardiac death; cerebral causes; trauma; mechanical asphyxia, including that caused by hanging; burns; carbon monoxide poisoning; hypothermia; sudden infant death syndrome; chemical poisoning/exogenous intoxication; and other, unspecified causes.

Kawasumi et al.^**(8)**^ and Sugawara et al.^**(10)**^ dedicated themselves to evaluating the PMCT findings in the bodies of drowning victims. In particular, both drew comparisons between fresh-water drowning and salt-water drowning. In most of the studies reviewed, CT scans were not performed on bodies that were in an advanced stage of putrefaction^**(3,^5^,^7^-^16^)**^. Some of the studies also excluded the bodies of children^**(^5^,^8^,^15^,^16^)**^, and others excluded cases of drowning in bathtubs on the grounds that a cardiovascular event, such as acute myocardial infarction, might represent an underlying pathology and contribute to the cause of death in that situation^**(13,^17^)**^.

In studies comparing the two methods, CT scans were performed prior to conventional autopsy, at intervals ranging from a few hours to 12 days postmortem.

### Imaging findings

The PMCT examinations were performed in 4-, 8-, 16-, 32-, 40-, 64-, 80-, and 160-slice scanners manufactured by GE Healthcare, Toshiba/Canon, Philips, Siemens, or Hitachi. In the studies reviewed, various protocols were used, some encompassing whole-body CT scans and others evaluating scans covering only the area from the skull to the pelvis, with different slice thicknesses, although most mentioned multiplanar reconstructions. No contrast medium was used in any of the PMCT scans acquired. The PMCT images were analyzed by radiologists or forensic pathologists.

Table 2 shows the main PMCT findings in drowning victims and the comparison with autopsy, for the studies that made such a comparison. Some of the studies used only autopsy to confirm the findings indicative of death by drowning but did not describe those findings or compare them with those of the PMCT scan^**(3,^4^,^10^,^11^,^13^,^14^,^16^,^17^)**^.

**Table 2:** —The main PMCT findings, in comparison with the main autopsy findings, in drowning deaths.

Authors	Main findings
Levy et al.^(^18^)^	**PMCT:** fluid in the mastoid air cells and paranasal sinuses, with hyperdense material in 25% of the bodies; fluid in the subglottic trachea, main bronchi, and secondary bronchi, with frothy fluid in 21% and dense material in 50%; crazy-paving pattern in the lungs; bilateral pleural effusion; gastric distention with dense sediment in 21%; and pulmonary edema and congestion in 100%. **Autopsy:** hyperdense material in the paranasal sinuses (in 25%), airways (in 50%), and stomach (in 21%) in the same proportion as on PMCT; frothy fluid in the airways, confirmed in only 19% of the cases; and features not identified on PMCT, including abrasions on the face and extremities, together with minor contusions, in 54%, as well as occult fractures in the skull base and in a rib.
Christe et al.^**(2)**^	**PMCT:** fluid in the paranasal sinuses, especially in the maxillary and sphenoid sinuses, in all cases; airway content in all cases (dense in 30% and liquid in 70%), mostly in the main bronchi; 40% of the tracheal volume filled with fluid; fluid in the pharynx in 80%; crazy-paving pattern in 60%; pulmonary edema in 50%; pleural effusion in 70%; hemodilution in the right atrium (density, 50 HU); and distention of the stomach and duodenum. **Autopsy:** Some airway content in all cases; 33% of the tracheal volume filled with fluid; fluid in the pharynx in 50%; crazy-paving pattern in 60%; pleural effusion in 80%; stomach distention similar to that seen on PMCT; and the classic finding of Paltauf spots in 70% of the cases.
Usui et al.^**(7)**^	**PMCT:** pulmonary findings classified as type 1 (in 34%), characterized by diffuse ground-glass opacities with interstitial thickening (crazy-paving pattern); type 2 (in 41%), characterized by poorly defined centrilobular nodules and diffuse ground-glass opacities; type 1 + type 2 (in 11%); type 3, characterized by consolidation (in 5%); type 4, characterized by emphysema, with or without fibrosis (in 4%); or type 5, defined as that which is unclassifiable (in 4%). **Autopsy:** pulmonary findings classified as type 1, characterized by marked edema (in 97% of the cases), the cut lung surface having a brick-red, protruding appearance, with a large amount of clear red fluid and white or bloody foam coming from each cut surface and bronchi; type 2, characterized by a protruding and reddish cut surface similar to that seen in type 1, but with less edema and no white foam; or type 4, characterized by pulmonary fibrosis.
Plaetsen et al.^**(12)**^	**PMCT:** fluid in the maxillary/ethmoid sinuses (in 98%), sphenoid sinuses (in 88%), and frontal sinuses (in 83%); fluid density of 19 HU and 20 HU in the right and left maxillary sinuses, respectively; fluid in the mastoid air cells in 12%, in the nasal cavities in 78% (frothy in 54%), in the nasopharynx in 98% (frothy in 40%), in the oropharynx in 95% (frothy in 40%), and in the trachea in 83%; ground-glass opacities in the lungs in 89% and consolidation in 11%, being diffuse in 54%, uneven (crazy-paving pattern) in 27%, and predominantly in the bases and posterior portions in 15%; pleural effusion in 71%, more commonly found in those drowned in salt water; depression of the right hemidiaphragm; blood density of 60 HU in the inferior vena cava and 59 HU in the right atrium (drowned in fresh water); pericardial effusion; fluid in one or two portions of the esophagus (in 91% and 49%, respectively); fluid distention of the stomach, with contents in multiple layers in 27%; and distention of the duodenum and jejunum (in 34% and 32%, respectively). **Autopsy:** Frothy fluid was less commonly observed, confirming the transient nature of this finding. Measurement of hemodilution in the right atrium and inferior vena cava was less reproducible and more time-consuming using the absorbent paper test.
Kawasumi et al.^**(8)**^	**PMCT:** fluid in the maxillary and sphenoid sinuses in victims of drowning in salt water and fresh water, with significantly greater density in those drowned in salt water than in those drowned in fresh water (47.28 HU vs. 32.56 HU). **Autopsy:** All cases were determined to be cases of wet drowning. None of the bodies showed fractures in the skull or facial bones.
Leth and Madsen^**(15)**^	**PMCT:** lung volume: 3,136 cm^3^; lung density: -1,073 HU; blood density in the right ventricle: 35 HU (drowning in fresh water) and 40 HU (drowning in salt water); and blood density in the pulmonary artery trunk: 52 HU (drowning in fresh water) and 54 HU (drowning in salt water). **Autopsy:** lung weight: 1,337 g; and lung density: 448 g/liter. Lung density was found to be was significantly lower in the bodies of drowning victims than in those of individuals who died from other causes.
Gotsmy et al.^**(19)**^	**PMCT:** gastric content layers in the following proportions: one layer in 41.8%, two in 40%, and three in 18.2%; and upper gastric content layers commonly more hypodense than the lower ones. When three layers were present, the upper layer always consisted of frothy material, whereas the lower layers contained denser components. **Autopsy:** gastric content layers in the following proportions: one layer in 50.9%, two in 34.5%, and three in 12.7%. In 28 of the 55 cases, a significant discrepancy was identified between the number of gastric content layers observed on PMCT and the number observed at autopsy.
Kakimoto et al.^**(5)**^	**PMCT:** The fluid volume in the maxillary sinus was significantly higher in drowning cases than in non-drowning cases. A total maxillary sinus fluid volume > 1.04 ml was more useful in indicating drowning than was a pleural effusion volume > 175 ml and a lung weight > 829 g. The combination of maxillary sinus fluid volume and pleural effusion volume was more effective in predicting drowning than was either index in isolation. Maxillary sinus fluid volume was less influenced by the postmortem interval, remaining valid for up to 1 week after death. **Autopsy:** The pleural effusion volume was significantly higher in drowning cases than in non-drowning cases. The mean lung weight among the cases of drowning was 1,093 g.
Heo et al.^**(9)**^	**PMCT:** sphenoid sinus fluid volume: mean, 2.7 ± 2.8 ml; and median, 1.65 ml. **Autopsy:** sphenoid sinus fluid volume: mean, 1.9 ± 1.7 ml; and median, 1.55 ml.

**Figure 2:**
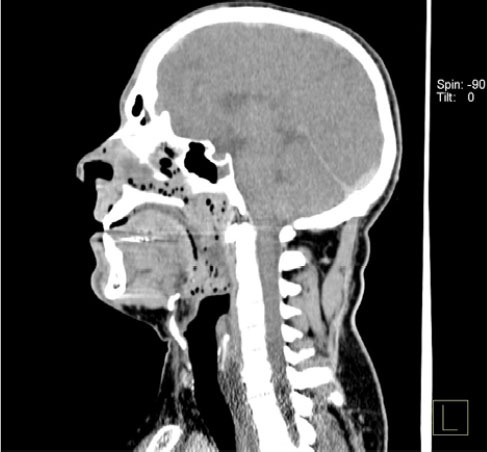
35-year-old male. PMCT scan of the skull and neck, with soft-tissue window settings and sagittal reconstruction, showing frothy fluid in the upper airways (nostrils, nasal cavity, sinuses, and pharynx).

**Figure 3:**
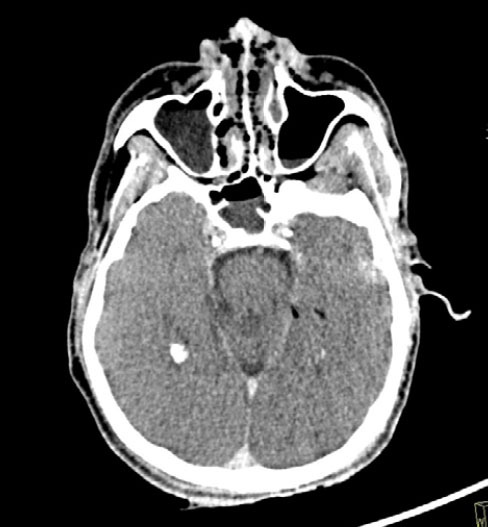
44-year-old male who died by drowning in fresh water (in a lake). Axial PMCT scan of the face, with soft-tissue window settings, showing frothy secretion in the nasal cavities and an air–fluid level in the paranasal sinuses, with greater fluid volume in the right maxillary sinus.

**Figure 4:**
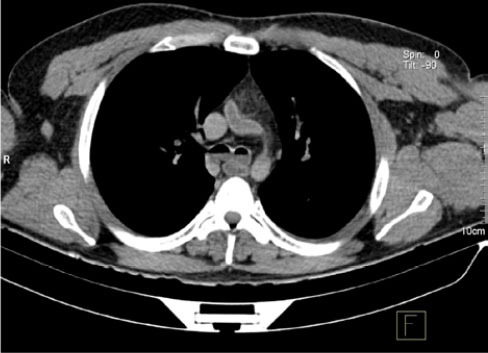
35-year-old male. Axial PMCT scan of the chest, with mediastinal window settings, showing an air–fluid level in the main bronchi.

**Figure 5:**
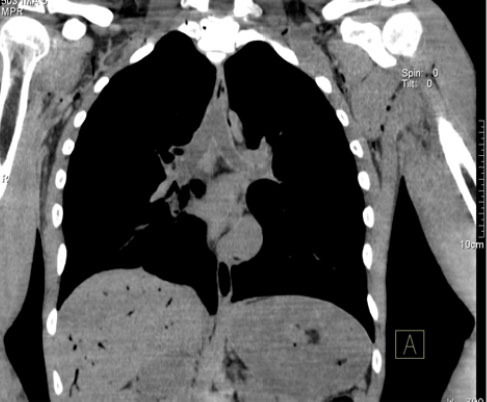
44-year-old male who died by drowning in fresh water (in a lake). Chest PMCT scan, with mediastinal window settings and coronal reconstruction, showing fluid in the trachea and main bronchi.

**Figure 6:**
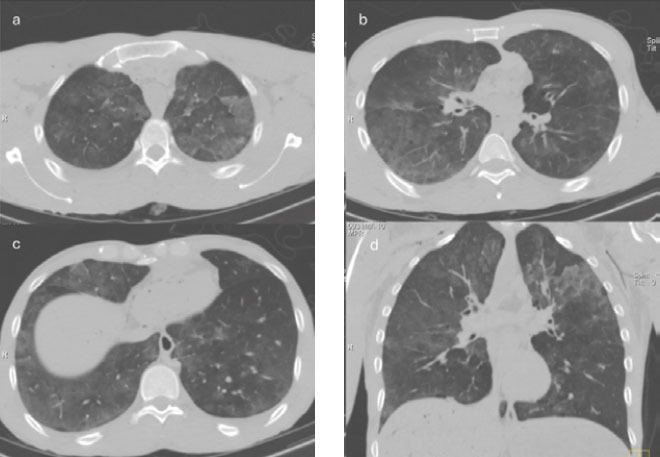
44-year-old male who died by drowning in fresh water (in a lake). A,B,C: Axial PMCT scan of the chest, with lung parenchyma window settings, showing images of the lungs at three levels, with bilateral, diffuse ground-glass opacities, some-times interspersed with interlobular and intralobular septal thickening, constituting areas of the crazy-paving pattern. D. Chest PMCT scan of the same body, with lung parenchyma window settings and coronal reconstruction, showing a predominance of the parenchymal changes described in the upper lung fields.

Of the findings described from the PMCT scans of drowning victims studied in this review, fluid in the paranasal sinuses was mentioned by 11 authors, fluid in the airways was mentioned by five, and fluid in the mastoid air cells was mentioned by three. Levy et al.^**(18)**^ described the presence of frothy fluid and high-attenuation sediment in the airways, representative of sand from salt water or iodine present in fresh water, as specific findings on the PMCT scans of drowning victims. Kawasumi et al.^**(4)**^ concluded that the presence of fluid in the maxillary and sphenoid sinuses was significantly associated with drowning, with a sensitivity of 97%, albeit with a specificity of only 35%, which allows us to state only that the absence of fluid in these sinuses probably excludes drowning. In addition, Kawasumi et al.^**(3)**^ showed that the volume of fluid in the maxillary or sphenoid sinuses was significantly greater in drowning cases than in non-drowning cases, whereas the fluid density was significantly lower. Kawasumi et al.^**(8)**^ suggested that the fluid density in the sinuses is a useful indicator to differentiate between drownings in salt water and drownings in fresh water. The cut-off value was 37.77 HU, with a negative predictive value of 91%. Illustrative examples are presented in Figures 2-5. Other PMCT findings in the bodies of drowning victims included the following: pulmonary parenchymal alterations (crazy-paving pattern, poorly defined centrilobular nodules with diffuse ground-glass opacities, and consolidation); pleural effusion; lowering of the right hemidiaphragm; hemodilution in the cardiac chambers; and fluid distention of the stomach and duodenum. Illustrative examples of parenchymal alterations are shown in Figure 6.

It is noteworthy that Usui et al.^**(7)**^ categorized the findings in the lungs of drowning victims into six types (described in Table 2). Of those, the type 4 cases did not demonstrate findings on PMCT similar to those considered typical of drowning at autopsy, suggesting that emphysema or fibrosis were preexisting in those lungs prior to death by drowning. Van Hoyweghen et al.^**(17)**^ showed that there was a statistically significant difference between cases of death by drowning and death from other causes in terms of the height of the right hemidiaphragm. Leth and Madsen^**(15)**^ found greater lung volume and lower lung density in drowning victims than in victims of death from other causes, both on PMCT and at autopsy. Gotsmy et al. ^**(19)**^ showed that liquid distention of the stomach was stratified into three layers of gastric content on PMCT scans of drowned bodies.

## DISCUSSION

This review highlighted the contribution of CT scans as a complementary tool in the diagnosis of death by drowning. Some characteristics were identified only on CT scans, whereas others were identified only at autopsy. In the studies reviewed, the most common PMCT finding in drowning victims was fluid in the paranasal sinuses, mastoid air cells, and airways. When that fluid is frothy and contains sediment with high attenuation, it becomes a specific finding. The absence of fluid in the sinuses on PMCT makes a drowning diagnosis unlikely. In addition, it has been shown that, in drowning deaths, the volume of fluid in the paranasal sinuses is greater and the density of that fluid is lower than in deaths from other causes. Regarding the type of water in which the drowning occurred (fresh or salt water), a cutoff of 37.77 HU for the density of fluid in the paranasal sinuses on PMCT has been suggested to characterize drowning in salt water; that is, drowning in salt water is unlikely when the fluid density is less than 37.77 HU. Notably, the authors of one study^**(16)**^ in this review, an animal study, warned that a finding of fluid in the paranasal sinuses should be interpreted with caution, given that it was not shown to distinguish between death by accidental drowning and submersion in water after death.

Other PMCT findings also highlighted in drowned bodies include the following: pulmonary parenchymal changes (crazy-paving pattern, poorly defined centri-lobular nodules with diffuse ground-glass opacities, and consolidation); pleural effusion; depression of the right hemidiaphragm; hemodilution in the cardiac chambers; and fluid distention of the stomach and duodenum, often with heterogeneous content. Regarding the last item, Gotsmy et al.^**(19)**^ suggested that the presence of three layers of gastric contents on PMCT is a strong forensic indication of death by drowning. Concerning hemo-dilution in the cardiac chambers, the present review documented that it was greater in the left chambers and in cases of drowning in fresh water. Physiologically, this can be explained by the fact that fresh water is hypotonic relative to plasma and is rapidly absorbed in the alveoli, passing into the pulmonary circulation and causing hemodilution. Regarding the pulmonary findings, Usui et al.^**(7)**^ stated that the phenomenon of aqueous emphysema established at autopsy might explain the appearance of poorly defined centrilobular nodules and diffuse ground-glass opacities on CT.

Paltauf spots, which are areas of subpleural hemorrhage measuring ≥ 2.0 cm, with irregular borders, that are light red in color, caused by the rupture of alveolar walls and blood capillaries. In one study^**(18)**^, the autopsy identified abrasions on the face and extremities, together with small contusions and occult fractures in the skull base and in a rib, none of which were identified on the PMCT scan.

On the basis of our review, the following PMCT findings can be considered indicative of death by drowning: fluid in the paranasal sinuses; pulmonary parenchymal changes (crazy-paving pattern, poorly defined centrilobular nodules with diffuse ground-glass opacities, and consolidation); pleural effusion; depression of the right hemidiaphragm; hemodilution in the cardiac chambers; and fluid distention of the stomach and duodenum. More specifically, the diagnostic criteria for death by drowning include distention of the stomach with heterogeneous contents, forming at least three layers; the diagnostic criteria for drowning in fresh water include greater hemodilution in the cardiac chambers; and the diagnostic criteria for drowning in salt water include fluid density in the paranasal sinuses with attenuation ≥ 37.77 HU. The absence of fluid in the maxillary and sphenoid sinuses is considered an exclusion criterion for death by drowning. The technical limitations reported in the studies reviewed here include small sample sizes and the difficulty in quantifying the volume and density of paranasal, pulmonary, or gastric fluids through the use of the methods employed to extract such material during conventional autopsy. Other limitations of PMCT include the absence of a well-established examination protocol and the scarcity of qualified professionals in the sub-specialty of forensic radiology, given that the accuracy of image interpretation depends on the completeness of the examination performed and the experience of the reader, highlighting the need for the standardization of protocols and ongoing training of professionals. The methodological heterogeneity observed in the 17 articles included in this review can be explained by broad timeframe considered (2007–2024), because the technology and the equipment have evolved, enabling the use of multidetector CT scanners and consequently improving the quality of the examination and shortening examination times, with thinner slices and faster reconstructions, for example. Although justified, that heterogeneity represents a potential limiting factor for the external validity of postmortem imaging findings. In comparison with conventional autopsy, PMCT also presents disadvantages in terms of its capacity to identify superficial lesions, soft-tissue injuries, and cardiovascular diseases.

Extrapolating to the reality in Brazil, we emphasize that there is a significant lack of studies on PMCT conducted in the country and an absence of national parameters. In addition, there is limited number of forensic medicine institutes with access to this technology for the evaluation of cases of drowning (a cause of violent death), currently present only in the cities of São Paulo, Brasília, Campo Grande, Belo Horizonte, Recife, and Goiânia.

## CONCLUSION

Pre-autopsy PMCT is a useful tool for visualization and documentation in the diagnosis of death by drowning. Although it can be considered a promising complementary method, it is as yet not a substitute for conventional autopsy.

Among the current technical and structural limitations to the full adoption of PMCT as an isolated post-mortem diagnostic alternative, we highlight the absence of a well-established examination protocol due to meth-odological heterogeneity and the scarcity of physicians qualified in the subspecialty of forensic radiology, which reduces the accuracy of the method. In addition, CT scanners are not widely available at forensic medicine centers around the world.

It is already certain that PMCT complements conventional autopsy for a more assertive diagnosis and will most likely allow an autopsy to be more localized and faster, as well as enabling review of the autopsy report, given that the stored images can be reevaluated at any time and do not constitute evidence that is subject to loss, unlike the body, which is lost to decomposition and the burial process.

## Data Availability

Not applicable
